# Ecosystem services of *Phragmites* in North America with emphasis on habitat functions

**DOI:** 10.1093/aobpla/plt008

**Published:** 2013-02-18

**Authors:** Erik Kiviat

**Affiliations:** Hudsonia, PO Box 5000, Annandale, NY 12504, USA

**Keywords:** Bio-energy, ecosystem services, habitat functions, invasive plants, management, methodology, non-native species, *Phragmites*

## Abstract

*Phragmites australis* (common reed) is widespread in North America, with native and non-native haplotypes. Many ecologists and wetland managers have considered *P. australis* a weed with little value to the native biota or human society. I document important ecosystem services of *Phragmites* including support for many common and rare species of plants and animals. This paper is based on an extensive review of the ecology and natural history literature, discussions with field workers, and observations in 13 US states and one Canadian province during the past 40 years. *Phragmites* sequesters nutrients, heavy metals and carbon, builds and stabilizes soils, and creates self-maintaining vegetation in urban and industrial areas where many plants do not thrive. These non-habitat ecosystem services are proportional to biomass and productivity. *Phragmites* was widely used by Native Americans for many purposes; the most important current direct use is for the treatment of wastes. Most of the knowledge of non-habitat ecosystem services is based on studies of *P. australis* haplotype M (an Old World haplotype). *Phragmites* also has habitat functions for many organisms. These functions depend on the characteristics of the landscape, habitat, *Phragmites* stand, species using *Phragmites* and life history element*.* The functions that *Phragmites* provides for many species are optimal at lower levels of *Phragmites* biomass and extent of stands. Old World *Phragmites*, contrary to many published statements, as well as North American native *Phragmites,* provide valuable ecosystem services including products for human use and habitat functions for other organisms. *Phragmites* stands may need management (e.g. thinning, fragmentation, containment or removal) to create or maintain suitable habitat for desired species of animals and plants.

## Abstract (precis)

In North America, *Phragmites australis* (common reed) has generally been regarded as a weed to be controlled. This paper shows that *Phragmites*-dominated vegetation provides important non-habitat ecosystem services (e.g., carbon sequestration, water quality maintenance) in proportion to its biomass, and many habitat functions for other organisms that vary depending on characteristics of the vegetation and surrounding landscape. *Phragmites* has both detrimental and beneficial functions; therefore decision-makers must clarify their management goals and understand the local situation. Extensive dense *Phragmites* may be managed to optimize ecosystem services by partial removal of biomass for a bioenergy feedstock.

## Introduction

*Phragmites australis* (common reed, hereinafter *Phragmites*) is widespread in North America (Clevering and Lissner 1999). Pre-Columbian Holocene fossils have been found in many localities (Rigg and Richardson 1938; additional citations in Kiviat and Hamilton 2001) and 40 000-year-old *Phragmites* was found in coprolites of the extinct *Nothrotheriops shastensis* (Shasta ground sloth) in an Arizona cave (Hansen 1978). Although *Phragmites* was evidently widespread in pre-Columbian North America, it is unclear how frequent or extensive it was in individual localities. *Phragmites* now occurs in patches in, or dominates the vegetation of, many fresh and brackish wetlands, littoral zones of lakes and ponds, disturbed wetlands, wet meadows, springs, seeps, ditches, swales and waste ground habitats such as wetland fill, mined areas and garbage landfill cover.

There are native and non-native haplotypes of *P. australis* in North America (Saltonstall 2002; Saltonstall *et al.* 2004). Excluding ‘Gulf Coast’ *Phragmites*, I refer to the native haplotypes as ‘native’ *Phragmites*, and the non-native haplotype M as ‘Old World’ *Phragmites* because it is widespread in Africa and Asia as well as Europe. (Native *Phragmites* was called *Phragmites australis americanus*, and Old World *Phragmites* was called *P. a. australis* by Saltonstall *et al.* 2004.) In North America, Old World *Phragmites* is most common in the northeastern states and progressively less common westward across the continent; native *Phragmites* is rare in the northeastern states, somewhat more common in the Middle Atlantic states, and most common in the western states (Saltonstall 2002). Gulf Coast *Phragmites* (*P. a. berlandieri*, *sensu* Saltonstall *et al.* 2004) occurs in peninsular Florida and on the Gulf Coast; it is a hybrid of Old World *P. australis* and *P. mauritianus* (Lambertini *et al.* 2012*a*). On morphological grounds, Ward (2010) asserted that Gulf Coast *Phragmites* of peninsular Florida is actually the widespread tropical species *Phragmites karka*. Lambertini *et al.* (2012*a*) discerned long-distance dispersal and hybridization of *Phragmites* on the US Gulf Coast and questioned the application of traditional species concepts to *Phragmites*. Genetic diversity is also high within all three kinds of *Phragmites*, and there is hybridization among the three entities (Lambertini *et al.* 2012*a*, *b*; Meyerson *et al.* 2012).

Several morphological and physiological features distinguish *Phragmites* from other wetland graminoids. *Phragmites* is large; it produces extensive colonies by means of underground rhizomes and ground-surface stolons, and the aerial shoots (culms) are 1–4+ m tall. Peak aboveground biomass in well-developed stands of the non-native haplotype M in the northeastern states can be 730–3700 g dry weight (dw) m^−2^ and exceeds the aboveground biomass of co-occurring marsh plants (Meyerson *et al.* 2000). One estimate of underground biomass from a New Jersey freshwater tidal marsh was 7180 g dw m^−2^, 6.7 times the peak aboveground biomass (Walker and Good 1976), and another estimate of underground biomass from a brackish tidal marsh in New Jersey was 1368 g dw m^−2^ (Windham 2001). In 17 studies, the density of living culms was 13–125 m^−2^ (Meyerson *et al.* 2000). In a freshwater tidal marsh on the Hudson River, standing (dead) mass in approximately April was similar to standing (dead plus live) mass in late June (Krause *et al.* 1997). Culms and leaves are rich in structural materials, including silica which stiffens these plant parts and helps to protect them from consumers and mechanical damage. In a European freshwater tidal marsh, *Phragmites* played an important role in cycling silicon (Struyf *et al.* 2007).

Although many dead culms stand for 2 years, *Phragmites* leaf blades decompose more rapidly; nonetheless, *Phragmites* litter may sequester nutrients and make them unavailable to other organisms (Meyerson *et al.* 2000). *Phragmites* marshes are capable of removing large amounts of pollutional nitrogen from surface waters (e.g. in Spain, González-Alcaraz *et al.* 2012). In the Chesapeake Bay region, Mozdzer *et al.* (2010) found that both *Phragmites* and *Spartina alterniflora* assimilated amino acids directly, and that urea nitrogen assimilation was greater in both native and Old World *Phragmites* than in *Spartina*. They also found affinity for dissolved organic nitrogen (DON) in decreasing order in native *Phragmites*, Old World *Phragmites* and *Spartina*, and estimated that as much as 47 % of *Phragmites* nitrogen demand could be satisfied by DON. *Phragmites* is effective in taking up nitrogen from the soil, and due to the greater biomass of *Phragmites* relative to co-occurring plants, aboveground standing stocks of nitrogen may be 2–3 times higher in *Phragmites* stands (Meyerson *et al.* 2000). In keeping with its high productivity, *Phragmites* efficiently oxygenates the rhizosphere during the growing season (Armstrong and Armstrong 1990). *Phragmites* has a C_3_ mechanism of carbon fixation and mature leaves have a structure consonant with that mechanism; however, the anatomy of young leaves is more like that of a C_4_ species (Antonielli *et al.* 2002).

Invasibility due to human alteration of hydrology, water quality, soils and vegetation plays an important role in the spread of haplotype M in North America (Kettenring *et al.* 2012). Seed viability of Old World *Phragmites* was low but variable in the Chesapeake Bay region, and some seeds were dormant at maturity whereas others were not (Kettenring and Whigham 2009). *Phragmites* seeds may require special conditions for germination and establishment. For example, falling water levels and exposed sandy bottoms were favourable for the spread of Old World *Phragmites* in the Great Lakes (Tulbure and Johnston 2010). Habitats created by *Castor canadensis* (American beaver), especially exposed bottoms of abandoned beaver ponds, are also suitable for the establishment of Old World *Phragmites* (E. Kiviat, unpubl. data).

Many ecologists and wetland managers in the USA and Canada have considered *P. australis* as a weed with little value to the native biota or human society (Meyerson *et al.* 2000, 2002; Kiviat 2010). Occasionally, ecologists have expressed the contrary view that reedbeds provide important habitat and other ecosystem services (e.g. Kane 2001*b*; Weis and Weis 2003). Here I show that *Phragmites* provides important ecosystem services, among which is support for common and rare elements of biodiversity including many species of native plants and animals. These habitat functions of *Phragmites* are linked to distinctive characteristics of the plant and are generally similar to habitat functions of *Phragmites* in the Old World. I also propose a new approach to managing *Phragmites* to optimize its habitat functions, potential harvest for products and other ecosystem services. It is important to present a detailed summary of habitat functions to create an accurate context for further research and management decisions.

## Methods

This paper is based on an extensive review of the ecology and natural history literature, discussions with many biologists and naturalists, 40 years of qualitative field observations and a series of quantitative field studies. I have studied *Phragmites* in 13 US states (New York, New Jersey, Maryland, Ohio, Connecticut and Massachusetts in the Northeast; Florida in the Southeast; New Mexico, Arizona, Utah, Colorado and southern California in the Southwest; North Dakota in the north-central region) and one Canadian province (Manitoba), as well as three countries in Europe (Czech Republic, Italy, the UK) and one in Africa (Botswana). In this paper, all observations are from North America unless identified otherwise. I refer to *Phragmites* stands or patches as ‘reedbeds’. In comparing the biota of reedbeds to alternate habitats, I have used abundance (density) of individual species and species richness because those metrics are most commonly available in the literature.

## Results and Discussion

The body of this paper addresses two categories of ecosystem service provided by *Phragmites*: non-habitat services, and habitat functions or biodiversity support.

### Non-habitat ecosystem services

Non-habitat ecosystem services (i.e. services other than biodiversity support) provided by *Phragmites* are listed in Table 1. Generally these services are proportional to biomass production because they are a function of physiological processes such as photosynthesis, nutrient uptake and transpiration.

**Table 1:** Non-habitat ecosystem services provided by North American *P. australis*. The classification of services follows MEA (2005).

Ecosystem service categories	*Phragmites* product or service	Source
*Provisioning services*		
Food	Seeds, sugar (historically and potentially)	Peterson (1977); Kiviat and Hamilton (2001)
Pharmaceuticals	Hallucinogen [dimethyltryptamine (DMT)]	Schultes *et al.* (2001); Website 1
Energy	Fuel pellets; potentially fuel bricks, methane, other fuels	R. Vaičekonytė *et al.*, Hudsonia, Annandale, New York, unpubl. data
Fibre	Roof thatch, fencing (Fig. 1), duckblind camouflage; craft paper, other crafts (Fig. 2); insulation	Harshberger and Burns (1919); Martin *et al.* (1957); Bell (1981); Ricciuti (1982); Johnsen (2003); J. Akenbach, Annapolis Thatching Co-op, Annapolis Royal, Nova Scotia, pers. comm.; F.X. Nsenga, TechnoPhrag, Montréal, Québec, pers. comm.
Ceremonial objects	Used in ceremonies by the Navajo, Arizona, and probably other groups of the southwestern states	C. Begay, Canyon de Chelly National Monument, Chinle, Arizona, pers. comm.
Miscellaneous products	Dried flower arrangements, other decorations and crafts; fishing poles	Kiviat (2010), unpubl. data
*Regulating services*		
Soil formation	Building and stabilizing soils	Windham and Lathrop (1999); Rooth and Stevenson (2000); Windham (2001); Rooth *et al.* (2003)
Carbon sequestration	Carbon sequestration	Windham (2001)
Climate regulation	Evapotranspirative ‘air conditioning’; high albedo	See text
Waste detoxification, contaminant sequestration, phytoremediation	Removal of contaminants from water or soil (largely experimental)	Windham *et al.* (2003); Ma, no date
Water quality maintenance	Removal of macronutrients from water	
Waste treatment	Dewatering sewage sludge; nitrogen and phosphorus removal from partially treated sewage	Kim *et al.* (1993); House *et al.* (1994); Burgoon *et al.* (1997); Saltman and Gallagher (1998)
Ecological restoration	Planted for restoration of springs by White Mountain Apache in the Southwest; stabilization and habitat development on inactive coal slurry impoundments	Nawrot and Yaich (1982); Jonathan Long, US Forest Service, pers. comm.
Crop pollination	Nest sites for bees	Bosch and Kemp (2001); Cane (2009)
*Supporting services*		
Primary production	Primary production	Whigham (1978); Meyerson *et al*. (2000)
Nutrient processing	Nutrient processing	Meyerson *et al.* (2000); Findlay *et al.* (2002, 2003)
*Cultural services*		
Cultural, intellectual, and spiritual inspiration	Literary and artistic inspiration (reed images in fiction, film, visual arts); ceremonial thatch for the Jewish Sukkot festival	Staab (1999); Wiener (2007); Wootton, no date; E. Kiviat, unpubl. data
Aesthetic	Maintenance-free spontaneous vegetation cover on urban and derelict lands; garden and landscape ornamental; left as a screen between industrial and residential areas	Geller (1972); Brown (1985); C. Detlefs, Rye City Naturalist, Rye, New York, pers. comm.; E. Kiviat, unpubl. data
Education, ecotourism	Education, ecotourism	
Scientific discovery	Research

**Figure 1.**
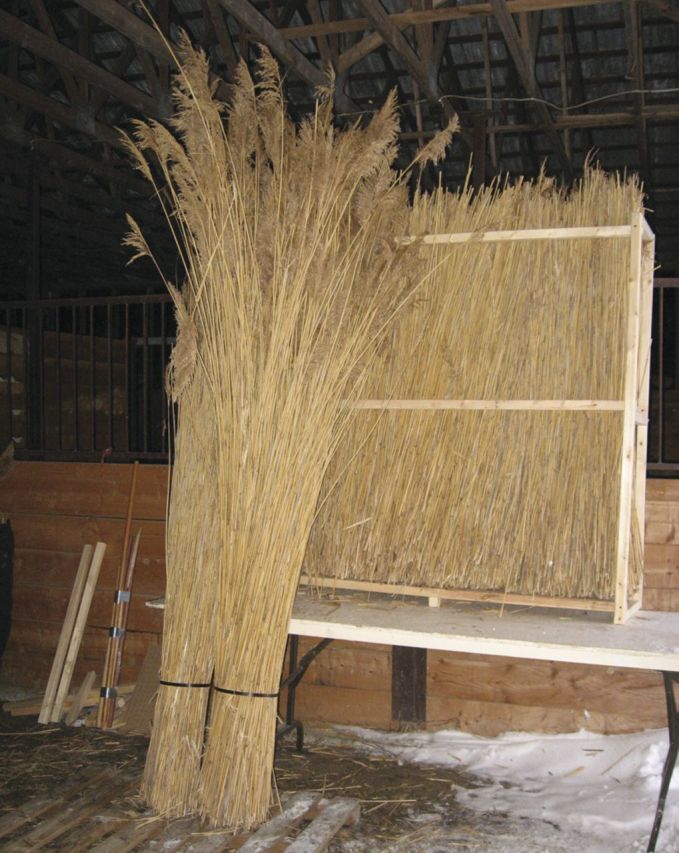
*Phragmites* fencing manufactured by TechnoPhrag, Montréal, Canada. Photograph by François Nsenga.

**Figure 2.**
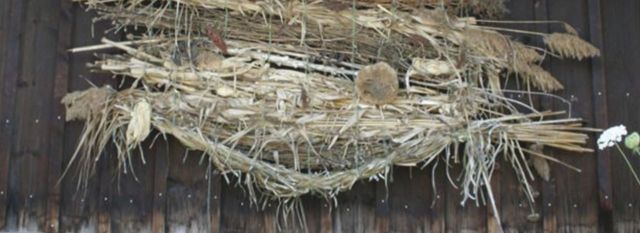
Detail of an art hanging containing *Phragmites* culms. Cornell Agroforestry Resource Center, Acra, New York.

Most of the *Phragmites* services shown in Table 1 are clearly beneficial to human society. However, biomass production and soil building, for example, can have detrimental effects on the habitats of certain organisms (Kiviat 2009*b*; see below) and may cause infilling of certain non-tidal wetlands to the point of reduction in water storage capacity.

#### Soil building and carbon sequestration

*Phragmites* builds and stabilizes tidal marsh soils, and stores carbon in litter and soils more effectively than *Spartina* spp. (Windham and Lathrop 1999; Rooth and Stevenson 2000; K. V. R. Schäfer, Rutgers University, Newark, NJ, pers. comm.). Thus *Phragmites* may protect tidal marshes from erosion associated with sea-level rise, as well as helping to mitigate global climate change. Soil building by *Phragmites* in tidal marshes appears to reduce micro-relief of the marsh surface and eliminate small pools used as a refuge at low tide by *Fundulus* (killifish) and other small nekton (Dibble and Meyerson 2012).

#### Products

There is direct use of *Phragmites* for roof thatch and other products in North America (Table 1). Thatching is practised on a small scale in Nova Scotia, Maryland, and occasionally elsewhere; however, the expense and lack of a tradition may inhibit expansion of this use. Commercial and home use of dried *Phragmites* for decoration in and outside the house is common. *Phragmites* is rarely planted in gardens or for landscaping (but see Urgo 2003), although superficially similar giant grasses such as *Cortaderia* (pampas grass) and *Miscanthus* (Eulalia) are often planted for ornament. Other uses of harvested *Phragmites* material (Table 1) appear to be uncommon or local. Excepting waste treatment, the level of extant direct use in North America is evidently lower than historic and prehistoric use of *Phragmites* by Native Americans, especially in the western USA (and northwestern Mexico; Kiviat and Hamilton 2001), and historic and contemporary use in parts of Europe (Hawke and José 1996) and the Tigris–Euphrates delta marshes of Iraq (Thesiger 1964).

#### Waste treatment

Clearly, the most important direct use of *Phragmites* in North America is in constructed systems for dewatering sludge from sewage treatment plants (e.g. Burgoon *et al.* 1997), and less frequently for removing nutrients from partially treated sewage (e.g. Gersberg *et al.* 1986). There are probably thousands of sludge-drying beds of variable size in the USA, and these are cost-effective and conserve energy that would otherwise be used in heat-drying of sludge. The high rates of transpiration of *Phragmites* and its ability to tolerate salt, metals and other pollutants make *Phragmites* suitable for drying sludge, and the efficient uptake of nutrients makes it suitable for polishing partially treated wastewater. *Phragmites* has also been used experimentally to dewater dredged material (Stout 1977).

#### Phytoremediation

The ability of *Phragmites* to take up metals and other toxic substances from soil and water, and its efficient aeration of the rhizosphere, have applications in phytoremediation (Weis and Weis 2004; Ma, no date). In brackish tidal marshes, Old World *Phragmites* was found to take up heavy metals from surface water and sequester them in biomass which would eventually be incorporated into marsh soil (Windham *et al.* 2003). Under some conditions, metals are retained in plaques on root surfaces (Mal and Narine 2004). Translocation of heavy metals from water to soil may make metals easier to remove from estuarine systems. This benefit may be counterbalanced by some loss of mercury from leaves to air (Kozuchowski and Johnson 1978; Windham *et al.* 2001).

#### Energy

The rapid growth and regrowth after cutting, and high level of biomass production, of *Phragmites* suggest a good feedstock for bioenergy. Indeed, *Phragmites* seems to be as good as *Panicum virgatum* (switchgrass) in this respect, and does not require the energy and fertilizer inputs that switchgrass does (R. Vaičekonytė, E. Kiviat, F. Nsenga and A. Ostfeld, Hudsonia, Annandale, NY, unpubl. data). We are studying *Phragmites* fuel pellets developed by François Nsenga (TechnoPhrag, Montréal, Canada), and the potential to produce pellets from *Phragmites* combined with other cellulosic waste products. Additionally, *Phragmites* (perhaps combined with other organic wastes) should be a good feedstock for methane generation by anaerobic digestion. Granéli (1984) suggested the use of *Phragmites* in Sweden for fuel pellets or other solid biofuels.

#### Other non-habitat services

High evapotranspiration from reedbeds, and their apparently high albedo, should ameliorate microclimates in urban areas and other regions subject to climatic warming by cooling the surroundings and reflecting the solar energy. Transpiration from *Phragmites* leaves was twice that from *S. alterniflora* (smooth cordgrass) leaves in a New Jersey tidal marsh (Windham *et al.* 2001). Living and dead *Phragmites* have been recommended for stabilizing and protecting levees and spoil banks (Headlee 1945; Stutzenbaker 1999). Other services are listed in Table 1.

### Habitat functions: how *Phragmites* supports biodiversity

Contrary to often-stated opinions, North American reedbeds support a great taxonomic, ecological and geographic diversity of native and non-native organisms (examples in Table 2; many more could be cited). These analyses require caution because many studies were qualitative, limited in spatial and temporal scope, or involved small samples.

**Table 2:** Some habitat functions of *Phragmites* in North America. Species (partial list) considered to be introduced (^i^) or native (^n^) so far as known.

*Phragmites* part	Use	Taxa	Source^a^
Inflorescence	Seeds—food	*Dolichonyx oryzivorus* (bobolink)^n^, Emberizidae (3 spp. of sparrows)^n^	Russak (1956); Lewis and Casagrande (1997); J. Bourque and R. Bourque, Brooklyn, New York; R.B. Renfrew, Vermont Center for Ecostudies, Norwich, Vermont; EK
	Nest material	*Passer domesticus* (house sparrow)^i^	J. Bourque and R. Bourque
	Shelter (and food)	Arthropods	Balme (2000); Tewksbury *et al.* (2002); Lambert (2005); Eichiner *et al.* (2011)
	Foraging site	*Archilochus colubris* (ruby-throated hummingbird)^n^	EK
Leaf blade	Food (Fig. 6)	*Simyra insularis* (Henry's marsh moth)^n^, *Poanes viator* (broad-winged skipper)^n^, *Ochlodes yuma* (Yuma skipper)^n^, *Orchelimum* (meadow katydid)^n^, *Hyalopterus pruni* (mealy plum aphid)^i^, *Branta canadensis* (Canada goose)^n^	Shapiro (1970); Scott *et al.* (1977); Balme (2000); Tewksbury *et al.* (2002); Lambert (2005); C. Bitler, Great Swamp National Wildlife Refuge, Basking Ridge, New Jersey; EK
	Nest material	*Cistothorus palustris* (marsh wren)^n^*, Reithrodontomys fulvescens* (fulvous harvest mouse)^n^	Svihla (1930); Kane (2001*a*)
	Shelter, substrate for egg cocoon	Salticidae (jumping spiders), other Araneae (spiders)	EK
	Foraging	Coccinellidae (lady beetles), Formicidae (ants)	EK
Leaf sheath	Shelter	Tight sheaths: *Chaetococcus phragmitis* (reed scale)^i^; loose sheaths: Araneae, Lepidoptera (moth larva)	Krause *et al.* (1997); Tewksbury *et al.* (2002); EK
	Foraging (eating reed scale)	*Poecile atricapillus* (black-capped chickadee)^n^, *P. carolina* (Carolina chickadee)^n^, *Agelaius phoeniceus* (red-winged blackbird)^n^	EK
Internode	Nest site	Apoidea (bees)^i,n^	Yurlina (1998)
	Shelter and food	Coleoptera (beetles), Diptera (flies)	Balme (2000); Tewksbury *et al.* (2002); Lambert (2005); EK
	Foraging	*Picoides pubescens* (downy woodpecker)^n^, other birds^n^	Lewis and Casagrande (1997); EK
Culm	Foraging	Coccinellidae, Araneae (web attachment)^n^	EK
	Support or perch	Vines (many spp.)^i,n^, Odonata (dragonflies and damselflies)^n^, Cicadoidea (cicada)^n^	EK
	Nest material	*Ardea alba* (great egret)^n^, *Nycticorax nycticorax* (black-crowned night-heron)^n^, *Plegadis falcinella* (glossy ibis)^n^, *Pandion haliaetus* (osprey)^n^, *Meleagris gallopavo* (wild turkey), among many others	Burger (1978); L. Benoit, University of Connecticut; EK
	Food	*Cuscuta* (dodder)^n^	EK
Leafy shoots	Food (esp. young shoots)	Livestock (horse, cow, sheep, goat)^i^, *Ondatra zibethicus* (common muskrat)^n^, *Sylvilagus floridanus* (eastern cottontail)^n^, *Sylvilagus* cf. *audubonii* (desert cottontail)^n^ (Fig. 3), *Lipara lucens* (gall fly)^i^	Ward (1942); Balme (2000); Tesauro (2001*a*, *b*); Tewksbury *et al.* (2002); Lambert (2005); Kiviat (2009*b*); EK
	Nest site	Birds (many spp.)^n^ (Fig. 4)	EK *et al.*, unpubl. data.
	Nest material	*Ursus americanus* (black bear)^n^, *Alligator mississippiensis* (American alligator)^n^	Svihla (1929); Hardiman (2001)
	Perch	Birds, *Calopteryx maculata* (ebony jewelwing damselfly)^n^, dragonflies^n^, etc.	EK
	Display perch	*Ammodramus maritimus* (seaside sparrow)^n^	Marshall and Reinert (1990)
	Foraging	*Cistothorus palustris*^n^, *Dendroica coronata* (yellow-rumped warbler)^n^, *Zonotrichia albicollis* (white-throated sparrow)^n^	EK
Rhizome	Food	*Rhizedra lutosa* (greater wainscot moth)^i^, *Chen caerulescens* (snow goose)^n^	Glazener (1946); Casagrande *et al.* (2003)
	Nest material	*Ondatra zibethicus*	Ward (1942); EK
Reedbed	Roosting	Ardeidae (herons, several spp.)^n^, *Buteo lagopus* (rough-legged hawk)^n^, *Circus cyaneus* (northern harrier)^n^, *Tyrannus tyrannus* (eastern kingbird)^n^, Hirundinidae (swallows, several spp.)^n^, *Turdus migratorius* (American robin)^n^, *Sturnus vulgaris* (European starling)^i^, Icteridae (blackbirds, several spp.)^n^	Meanley (1965, 1971, 1993); Bosakowski (1983); Buchsbaum (1991); Kane (2001*a*, *b*); Petersen (2001); Kiviat and Talmage (2006); Iliff and Lovitch (2007)
	Nest site	*Castor canadensis* (American beaver)^n^, *Ondatra zibethicus*^n^, *Ixobrychus exilis* (least bittern)^n^, other Ardeidae^n^, Threskiornithidae (ibises, 3 spp.)^n^, *Anas platyrhynchos* (mallard)^n^, *Aythya americana* (redhead)^n^, *A. valisineria* (canvasback)^n^, *A. affinis* (lesser scaup)^n^, *Oxyura jamaicensis* (ruddy duck)^n^, *Circus cyaneus*^n^, *Rallus longirostris yumanensis* (Yuma clapper rail)^n^, *Gallinula chloropus* (common moorhen)^n^, *Larus argenteus* (herring gull)^n^, *Larus atricilla* (laughing gull)^n^, *Agelaius phoeniceus*^n^, *Quiscalus quiscula* (common grackle)^n^, many other birds^n^	Hecht (1951); Weller (1961); Burger (1977); Burger and Shisler (1980); Anderson and Ohmart (1985); Lewis and Casagrande (1997); Kane (2001*a*, *b*); Peer *et al.* (2001); Hinojosa-Huerta *et al.* (2004); EK
	Foraging	*Picoides pubescens*^n^, *Sayornis phoebe* (eastern phoebe)^n^, *Poecile atricapillus*^n^, *Dendroica petechia* (yellow warbler)^n^, *Cistothorus palustris*^n^	Ward (1942); Kane (2001*a*); EK
	Shelter (escape cover, etc.)	*Odocoileus virginianus* (white-tailed deer)^n^, *Sylvilagus* cf. *audubonii*)^n^, *Aythya americana*^n^ (duckling escape cover)	Hecht (1951); Naugle (1997); Smith (1997); Meyer (2003)
	Shelter from weather	Anatidae (waterfowl)^n^, *Passer domesticus* (house sparrow)^i^, *Quiscalus quiscula*^n^, *Odocoileus virginianus*^n^	Smith (1997), Kane (2001*a*, *b*); EK
	Shelter	Bryophyta (mosses, many spp.)^n^, Marchantiophyta (few spp. of liverworts)^n^, *Limosella subulata* (mudwort)^n^*, Lilaeopsis chinensis* (eastern lilaeopsis)^n^, *Cardamine longii* (Long's bittercress)^n^	Ward (1942); Barbour and Kiviat (2007); G. Stevens, Hudsonia, Annandale, New York; EK
	Occurrence (type of use not described)	*Anaxyrus microscaphus* (Arizona toad)^n^, *Chelydra serpentina* (common snapping turtle)^n^, *Glyptemys muhlenbergii* (bog turtle)^n^, *Thamnophis sirtalis* (eastern garter snake)^n^, *Crotalus viridis helleri* (southern Pacific rattlesnake)^n^, *Nycticorax nycticorax*, *Botaurus lentiginosus* (American bittern)^n^, *Phasianus colchicus* (ring-necked pheasant)^i^, *Asio flammeus* (short-eared owl)^n^, *Empidonax traillii* (willow flycatcher)^n^, *Corvus brachyrhynchos* (American crow)^n^, *Cyanocitta cristata* (blue jay)^n^, *Baeolophus bicolor* (tufted titmouse)^n^, *Passer domesticus* (house sparrow)^i^, *Dendroica petechia*^n^, *Geothlypas trichas* (common yellowthroat)^n^, *Carduelis tristis* (American goldfinch)^n^, *Cardinalis cardinalis* (northern cardinal)^n^, *Passerculus sandwichensis* (savannah sparrow)^n^, *Melospiza melodia* (song sparrow)^n^, *M. georgiana* (swamp sparrow)^n^, *Peromyscus leucopus* (white-footed mouse)^n^, *Zapus hudsonius* (meadow jumping mouse)^n^, *Microtus pennsylvanicus* (meadow vole)^n^, *Ondatra zibethicus*^n^, *Mus musculus* (house mouse)^i^, *Canis latrans* (eastern coyote)^n^, *Odocoileus hemionus* (mule deer)^n^, many others	Hecht (1951); Holland and Smith (1980); Buchsbaum (1991); Buchsbaum and Hall (1991); Lewis and Casagrande (1997); Kane (2001*a*); Whitlock (2002); Meyer (2003); McGlynn (2006); M.W. Klemens, American Museum of Natural History, New York; EK
Pools and creeks within reedbeds, reed-bordered ditches	Occurrence, foraging, moulting	*Anaxyrus americanus* (American toad)^n^, *Lithobates* sp. (leopard frog)^n^, *Phalacrocorax auritus* (double-crested cormorant)^n^, *Anas rubripes* (American black duck)^n^, *Anas platyrhynchos* (mallard)^n^, Anatidae (other ducks)^n^, *Fulica americana* (American coot)^n^, *Gallinula chloropus* (common moorhen)^n^, *Tringa melanoleuca* (greater yellowlegs)^n^, *Sterna forsteri* (Forster's tern)^n^, *Sternula antillarum* (least tern)^n^	Ward (1942); Buchsbaum (1991); Buchsbaum and Hall (1991); Kane (2001*a*, *b*); Kiviat (2011); EK
Whole plant or unspecified	Nest material	*Alligator mississippiensis* (American alligator)^n^, *Egretta thula^n^, Nyctanassa violacea* (yellow-crowned night-heron)^n^*, Plegadis falcinella^n^, Branta canadensis*^n^, *Circus cyaneus*^n^, *Ondatra zibethicus*^n^, *Castor canadensis*^n^	Svihla (1929); Klopman (1958); Ciaranca *et al.* (1997); Hayes-Odum and Dixon (1997); Kane (2001*a*); R. Kane; EK
Culm base	Substrate Shelter	Bryophyta (many spp. of mosses)^n^, Marchantiophyta (few spp. of liverworts)^n^ Araneae^n^ in hollow broken internode	G. Stevens, Hudsonia, Annandale, New York; EK
Litter, detritus	Substrate, food	Acari (mites), Collembola (springtails), Insecta (insects)	Hudson (1994); Talley and Levin (2001); Kiviat and Talmage (2006)

^a^EK, Erik Kiviat, unpubl. data; a name and affiliation without a year signify a personal communication.

**Figure 3.**
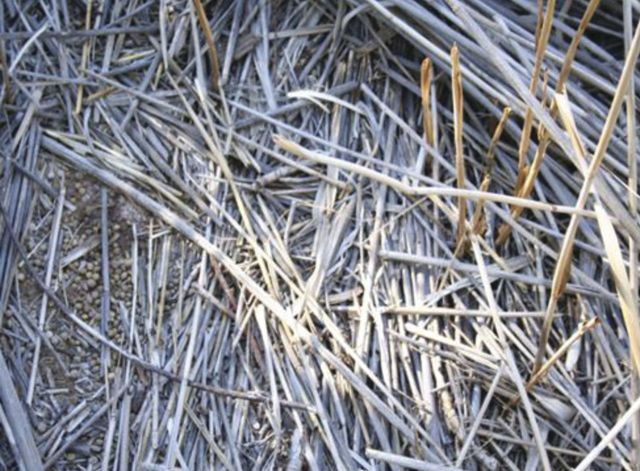
*Sylvilagus* cf. *audubonii* (desert cottontail rabbit) scats on the ground in lower left and *Phragmites* culm stumps cut at an angle in upper right. Photograph by Erik Kiviat.

**Figure 4.**
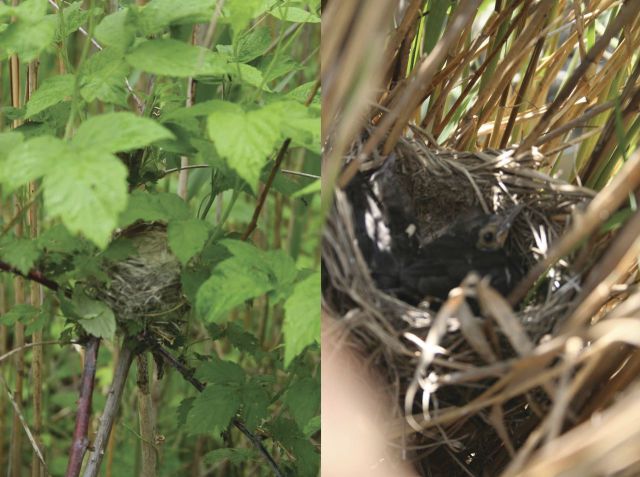
Birds nesting in *Phragmites*. Left: *Dendroica petechia* (yellow warbler) nest in *Rubus occidentalis* surrounded by Old World *P. australis*. Dry habitat on dredged material island, Hudson River. Right: *Quiscalus quiscula* (common grackle) nest in Old World *P. australis*. Freshwater tidal marsh, Hudson River. Photographs by Erik Kiviat.

**Figure 5.**
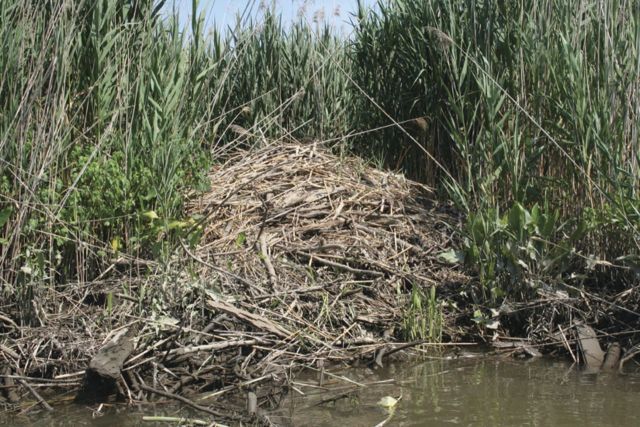
Beaver lodge partially constructed of—and surrounded by—Old World *P. australis*, on the bank of a large, tidal creek. An admixture of other plants is visible in the reedbed edge. Hudson River, New York. Photograph by Erik Kiviat.

**Figure 6.**
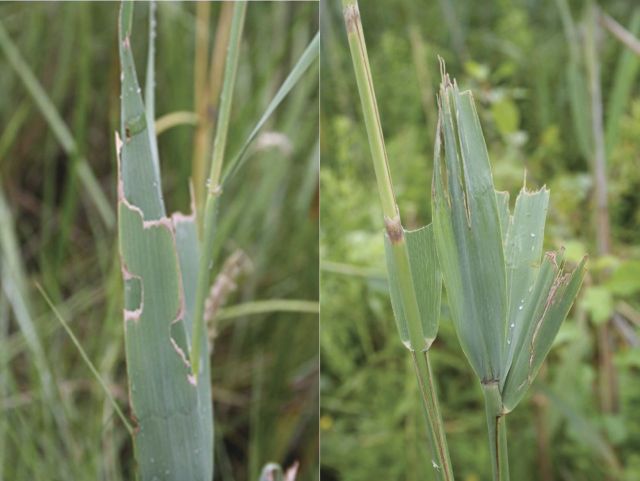
Left: insect-grazed leaves of Old World *P. australis*. Randall's Island, New York City, 20 September 2011. Right: cattle-grazed leaves of Old World *P. australis*, Amenia, New York, 19 July 2012. Photographs by Erik Kiviat.

#### *Phragmites* as food

**Figure 7.**
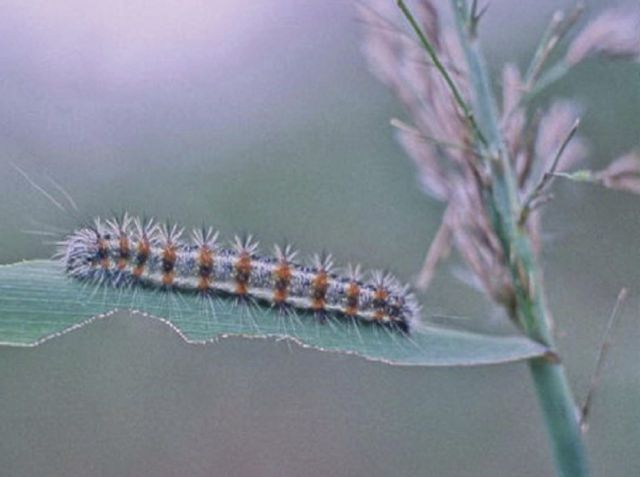
*Simyra insularis* (Noctuidae; Henry's marsh moth) larva grazing Old World *P. australis* leaf blade, New Jersey Meadowlands. Photograph by Erik Kiviat.

Various insects feed on *Phragmites* (Balme 2000; Tewksbury *et al.*, 2002; Lambert 2005; E. Kiviat, unpubl. data); many of these are believed to be non-native (Balme 2000). However, most studies of *Phragmites* insects have been in the eastern states and there are probably many insects associated with western *Phragmites* that remain to be documented. Insects include endophagous stem-feeders, leaf chewers, sap suckers, gall makers and a rhizome feeder. Usually, insect feeding does not cause significant damage; Balme (2000) found the greater wainscot moth *Rhizedra lutosa* causing minor damage in Rhode Island. On one occasion I found larvae of *Simyra insularis* (Noctuidae; Henry's marsh moth), a native, generalist feeder, heavily grazing *Phragmites* leaf blades where it grew sparsely among *Calamagrostis canadensis* (bluejoint grass), but not in the adjoining dense *Phragmites* stands (Fig. 7).

The non-native sap-feeding *Chaetococcus phragmitis* (reed scale) that is sessile beneath lower leaf sheaths may be widespread and abundant, at least in Old World *Phragmites*. Krause *et al.* (1997) found late-winter biomass of adults as high as 1 g dw m^−2^ in a freshwater tidal marsh on the Hudson River. I have frequently seen songbirds opening leaf sheaths and consuming the scale insects, especially in winter, in the northeastern states. Birds also commonly peck holes in *Phragmites* internodes and eat insects living within.

*Hyalopterus pruni* (mealy plum aphid) is widespread and abundant in North America (Balme 2000; Lambert 2005). This aphid alternates generations between *Phragmites* in summer and *Prunus* spp. (cherries, etc.) in winter; it is a pest of prune (*Prunus domestica*) orchards in California (Latham and Mills 2012). Although birds apparently do not feed on mealy plum aphid, Coccinellidae (lady beetles) are often present and presumably feed on the aphids.

*Ondatra zibethicus* (common muskrat) is the most important native vertebrate consumer of *Phragmites*. Muskrats feed on young shoots and rhizomes, and also cut mature culms for lodge construction. Several studies in different regions of North America have found *Phragmites* ranking from high to low among other plant species in the muskrat diet (Bellrose 1950; Paradiso 1969). Muskrats may use *Phragmites* intensively, depending on the availability of more ‘preferred’ foods such as *Typha* (cattail) and *Scirpus* (bulrush; Butler 1940; McCabe 1982). For example, Butler (1940) listed *Phragmites* as the fourth of 13 plant taxa in the muskrat diet in Manitoba; McCabe (1982) found *Phragmites* a close second to *Scirpus* in Utah; *Phragmites* was an important summer food in the north-central states (Errington 1941); in Maryland tidal marshes *Typha* and *Scirpus* were most important but *Phragmites* was ‘a favourite food, grows in beds of limited distribution, in which muskrats are always found’ (Smith 1938); and feeding on *Phragmites* in Louisiana coastal marshes varied according to marsh type (O'Neil 1949). Nonetheless, Ward (1942), Lynch *et al.* (1947) and Martin *et al.* (1957) considered *Phragmites* to be a low-quality or uncommonly eaten food. In Louisiana, 10 % or less of the muskrat activity (including feeding) was associated with *Phragmites* (O'Neil 1949), and muskrat use of *Phragmites* stands in Connecticut tidal marshes was consistently low (Benoit and Askins 1999). Muskrats may be abundant in habitats where *Phragmites* is highly dominant, as at times and places in the New Jersey Meadowlands (E. Kiviat, unpubl. data). *Castor canadensis* (American beaver) also uses *Phragmites* for construction and perhaps eats it as well, but possibly less so than the muskrat.

*Sylvilagus* spp. (cottontail rabbits) at times cut many *Phragmites* shoots for food (Balme 2000; E. Kiviat, unpubl. data). Balme (2000) found extensive clipping of culms by *Sylvilagus floridanus* (eastern cottontail) in experimental *Phragmites* plots in Rhode Island. I found extensive clipping by *S. floridanus* at a lakeside wet meadow in Rockland County, New York, in 2011. In 2006 I observed much use of *Phragmites* stands (clipping of culms, shelter) by *S.* cf. *audubonii* (desert cottontail) in the Southwest. Domestic livestock (horses, cattle, goats, sheep) graze *Phragmites*, especially young shoots in spring, and have caused *Phragmites* declines in some cases (Kiviat and Hamilton 2001). Spatial patterns of reedbeds in relation to fences of livestock pastures in New York suggest that livestock inhibition of *Phragmites* is common. *Odocoileus virginianus* (white-tailed deer) may graze *Phragmites* in Louisiana but it is not a major food (Self *et al.* 1975). *Branta canadensis* (Canada goose) grazes *Phragmites* leaf blades, especially in urban marshes of the New York City area, but does not seem to do much damage (E. Kiviat, unpubl. data). *Chen caerulescens* (snow goose) feeds on *Phragmites* rhizomes in Gulf Coast marshes (Glazener 1946).

Dead *Phragmites* material (litter, detritus) provides food as well. Fungi and other microbes growing on decomposing wetland plants support detritivorous invertebrates (Gulis *et al.* 2006) and provide the basis for wetland food webs that are often more important than those based on herbivory. Most of the macroinvertebrates found in reedbed litter and soil (see Table 3) are probably deriving nutrition from dead *Phragmites* and associated microbes. Food webs based on *Phragmites* detritus, alone or as a significant portion of mixtures with other carbon sources, can support important fish populations (Wainwright *et al.* 2000; Weinstein *et al.* 2000) and therefore higher-order consumers that presumably include certain invertebrates, turtles, snakes, many kinds of birds, and mammals.

**Table 3:** Comparisons of the numbers of individuals (density) or species (richness) of macroinvertebrates in *Phragmites* and alternate habitats. *Ca*, *Carex* (sedges); *Ls*, *Lythrum salicaria* (purple loosestrife); *SpA*, *Spartina alterniflora* (smooth cordgrass); *SpP*, *Spartina patens* (saltmeadow cordgrass) and associated short graminoids; *Sc*, *Scirpus cyperinus* (wool-grass); *Ty*, *Typha* (cattails); *W*, woody vegetation; N, non-tidal.

Invertebrate	Comparison	Alternate habitat	Source
Benthos invertebrates, litter invertebrates, or both	Density or richness: in 4 studies equal to or nearly so, in 1 study greater than, and in 4 studies less than in alternate habitat	*SpA, SpP, Ty; Sc* (N)	Fell *et al*. (1998); Angradi *et al.* (2001); Talley and Levin (2001); Warren *et al*. (2001); Osgood *et al.* (2003); Posey *et al.* (2003); Yuhas *et al.* (2005); Kiviat and Talmage (2006); McGlynn (2006); Kennedy (2008)
*Geukensia demise* (ribbed mussel)	Density equal	*SpA*	McClary (2004)
Gastropoda (snails)	Density, diversity, biomass equal to or greater than in alternate habitat	*Ty* (N)	Back (2010)
Nektonic crustaceans	Density or richness: in 3 studies equal to, in 1 study greater than, and in 2 studies less than in alternate habitat	*SpA, Ty*	Able and Hagan (2000); Meyer *et al.* (2001); Fell *et al.* (2003); Osgood *et al.* (2003); Buchsbaum *et al.* (2006); Fell *et al.* (2006)
Epifauna	Richness less than in alternate habitat	*SpA*	Robertson and Weis (2005)
Terrestrial arthropods	Density and richness less than (1 study) and biomass greater than (1 study) in alternate habitat	*SpA; Ty* and *Ls*	Krause *et al.* (1997); Gratton and Denno (2005)
*Dermestes nidum* (hide beetle)	Abundance equal	*W*^a^	Parsons *et al.* (2009)
Flying insects	Abundance less than in alternate habitat	*Ca* (N)	Garcia (1998)

^a^Hide beetles in nests of Ardeidae (herons) and Threskiornithidae (ibises).

#### *Phragmites* as shelter, substrate and habitat (Fig. 8)

Reedbeds in which *Phragmites* is highly dominant are often called ‘monotypic’, ‘pure’, or ‘monodominant’. There may be an absence of other vascular plants at the scale of 1 m^2^ but rarely is this true at a larger scale, e.g. 100 m^2^. In many cases, stands of robust, dense *Phragmites* have smaller associated plants in the outer 1 m of reedbed edge, but support few species or individuals of other vascular plant species, or those other plants are stunted, in the stand interiors. Frequent associates in reedbed interiors include *Peltandra virginica* (arrow arum) and *Impatiens capensis* (orange jewelweed) in fresh water, and *Atriplex prostrata* (*A. patula* var. *hastata*; orache) in brackish water. Occasional individuals of larger woody or suffrutescent species such as *Sambucus nigra* ssp. c*anadensis* (common elderberry), *Ailanthus altissima* (tree-of-heaven), or *Hibiscus moscheutos* (swamp rose mallow) may also occur; in some cases these plants may have been present before reedbed development. *Betula pumila* (swamp birch), a shrub or small tree, was present in a Massachusetts fen before *Phragmites* colonization, and when *Phragmites* was removed, *B. pumila* grew taller (J. M. Toro, Native Habitat Restoration, Stockbridge, MA, pers. comm.). Similarly, *Taxodium distichum* (bald-cypress) and *Cephalanthus occidentalis* (buttonbush) planted in a created non-tidal wetland in Beltsville, Maryland, persisted despite colonization by a dense stand of *Phragmites* (A. H. Baldwin, University of Maryland, USA, pers. comm.). Keller (2000) found the diversity of associated plants to be lower in *Phragmites* than in *Lythrum salicaria* (purple loosestrife) or *Typha*. Meyerson *et al.* (2000) also compiled several studies showing lower vascular plant diversity in reedbeds.

**Figure 8.**
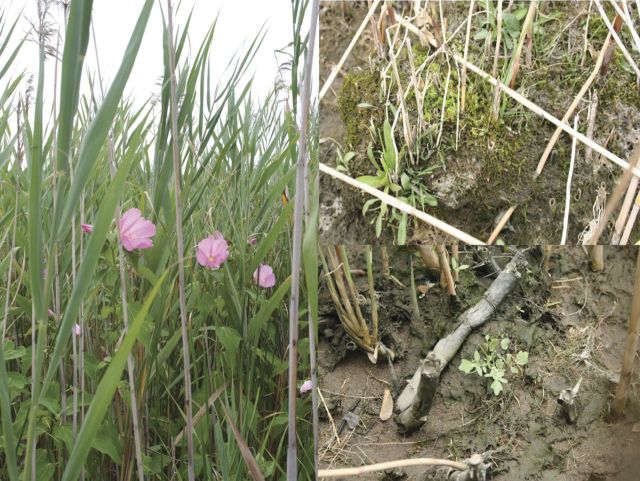
Other plants associated with reedbeds. Left: *Hibiscus moscheutos* (swamp rose mallow), a common large suffrutescent associate of Old World *P. australis* in East Coast tidal marshes and formerly tidal marshes. Upper right: mosses beneath sparse Old World *P. australis* on freshwater tidal shore, Hudson River. Lower right: *Cardamine longii* (Long's bittercress), a rare plant, beneath sparse Old World *P. australis* on the freshwater tidal shore, Hudson River. Photographs by Erik Kiviat.

Reedbeds can be dense, with *Phragmites* highly dominant, or sparse with other species admixed. For example, in September 2000, I found 18 species of associated vascular plants (three herbaceous and one woody vines, two shrubs, two suffrutescent herbs, two ferns, and eight other herbs) in the interior of a reedbed that had been harvested annually and occasionally burned in the New Jersey Meadowlands (E. Kiviat, unpubl. data). These associated species were sparse and occurred just outside the most recently harvested area. Reedbeds that are sparse, deeply flooded, or subject to high hydrodynamic energy (e.g. shorelines of open tidal waters) may support a greater diversity of vascular plants in edges. The occurrence of rare vascular plants and mosses in the interiors or edges of reedbeds under some circumstances suggests that *Phragmites* is facilitating the associated species by ameliorating harsh environmental conditions. Some of the cases I have observed are in relatively high-energy (wave-washed) tidal shores where sparse reedbeds appeared to be physically sheltering smaller plants of other species or maintaining favourable substrates against wave erosion. At Jamaica Bay Wildlife Refuge in New York City, *Platanthera lacera* (ragged fringed orchid), a regionally rare species, was found beneath mixed upland stands of *Phragmites* and *Betula populifolia* (grey birch), and nowhere else (D. Taft, U.S. National Park Service, New York, NY, pers. comm.). On the Hudson River, three rare native species, *Limosella subulata* (mudwort) and *Lilaeopsis chinensis* (eastern lilaeopsis) in brackish tidal wetlands, and *Cardamine longii* (Long's bittercress) in fresh-tidal wetlands, occur in reedbed edges where the *Phragmites* may be facilitating these small plants by providing physical shelter, stabilizing the sediments, or oxygenating the soil (the last phenomenon was suggested as a process by which *Phragmites* facilitated plants less tolerant to soil hypoxia; Callaway 1995).

Vines, both woody and herbaceous species, use *Phragmites* for support. Vines are especially frequent and sometimes constitute considerable phytomass at the upland edges of reedbeds and on channel banks where the substrate may be slightly higher. *Cuscuta* (dodder) occasionally parasitizes *Phragmites*; all other vines are non-parasitic. Certain robust woody vines that ordinarily use woody plants or permanent structures such as fences for support evidently are able to reach from old overwintered *Phragmites* culms to new shoots of the current year. I have documented >30 species of vines, half native and half non-native, using *Phragmites* as the host (E. Kiviat, unpubl. data). Vines modify reedbed architecture and provide additional food resources for animals.

Diverse mosses and a few liverworts occur beneath reedbed edges and interiors on soil or culm bases (Barbour and Kiviat 2007; G. Stevens, Hudsonia, Annandale, NY, pers. comm.; E. Kiviat, unpubl. data). Bryophytes appear to be more abundant and diverse beneath *Phragmites* where it grows sparsely and the substrate is wet but not long-flooded. A rare species in New York, the moss *Philonotis muhlenbergii*, was found beneath *Phragmites* on a Hudson River island (Barbour and Kiviat 2007). Algae colonize the lower portions of culms. Epiphyton (algae, particularly diatoms) was similar in *Phragmites* and *Typha* in an Ohio marsh (Back 2010).

Reedbeds may retain ice and remain cooler than their surroundings in spring (Meyerson *et al.* 2000). The resulting cool microclimate may inhibit some biota. Possibly some of these cool reedbeds shelter species near their southern range limits that require cool habitats.

The greater height of *Phragmites* compared with other wetland herbs is a resource for certain species. Although the nests of *Ammodramus maritimus* (seaside sparrow) were placed low in native graminoids in Massachusetts, the birds most often sang from *Phragmites* or a shrub [*Iva frutescens* (marsh-elder); Marshall and Reinert 1990]. *Phragmites* located at higher substrate elevations in or near marshes, and perhaps the robust nature of the reedbed itself, can provide shelter from higher than normal tides or floods, as evidenced by nesting *Larus atricilla* (laughing gull) in New Jersey (Burger and Shisler 1980).

Particular features of reedbeds attract birds in many instances. Anatinae (dabbling ducks) loafed on cattle-trampled reedbeds at the Delta Marshes, Manitoba (Sowls 1955). Small, reed-bordered channels were used by ducks during bad weather in the New Jersey Meadowlands [R. Kane, New Jersey Audubon Society (retired), Bernardsville, NJ, pers. comm.]. Reedbeds, especially those with standing water, attract large numbers of roosting songbirds, as reported in published studies and qualitative observations (Table 2); in one example, there was a peak of 40 000 *Dolichonyx oryzivorus* (bobolink; Iliff and Lovitch 2007). In the Delta Marshes of Manitoba, where native *Phragmites* is a dominant species, *Circus cyaneus* (northern harrier) nested in the edges between *Phragmites* and *Scholochloa festucacea* (whitetop grass). *Phragmites* was the most abundant plant in the vicinity of five nests (Hecht 1951).

Few data are available regarding *Phragmites* support of amphibians and reptiles, although various species have been found in reedbeds (Table 2). Under certain circumstances, reptiles appear to be using reedbeds for overwintering or thermoregulation (E. Kiviat, unpubl. data). *Storeria dekayi dekayi* (northern brown snake) individuals have been found beneath small piles of recently cut *Phragmites* culms in a non-tidal marsh restoration site in New York City (V. Ruzicka, Randall's Island Park Alliance, New York, NY, pers. comm.).

#### *Phragmites* as nest material

Many birds use *Phragmites* culm, leaf, or inflorescence material in their nests. Common muskrat and American beaver use culm and rhizome material in lodge construction. No information is available comparing *Phragmites* with alternate materials. Muskrat and beaver lodge construction may disperse living fragments of rhizome or culm base because some of the nest material remains wet.

#### *Phragmites* as a buffer

The tall, dense, resilient masses of *Phragmites* often provide a buffer between human activities or cattle grazing and wetland wildlife (Ward 1942; Buchsbaum 1991). *Phragmites* screens out some of the noise and visual disturbances. Dense woody thickets can provide the same function, although reedbeds often occur at marsh edges in urban areas and other places that lack dense shrubs or trees. Reedbeds also buffer other organisms from winds. On Lake Poygan, Wisconsin, artificial nesting platforms for *Sterna fosteri* (Foster's tern) were anchored in reedbeds to provide shelter for the nests (Mossman *et al.* 1988). Dense reedbeds are noisy when a human or a predator forces its way through the *Phragmites* culms; this warns smaller animals hiding or roosting in the reedbeds.

#### Habitat combinations

Mobile animals, such as birds, many mammals, and strongly flying insects, commonly use combinations of habitats to acquire all the resources they need. A reedbed can support one type of activity by a species while an adjacent or nearby alternate habitat can support another type of activity. In Marshlands Sanctuary (New York), *Rallus longirostris* (clapper rail) nested in a narrow fringe of *Phragmites* at the upland edge of a brackish tidal marsh, and foraged in the adjacent *S. alterniflora* at a slightly lower elevation in the marsh (A. Beal, Westchester County Department of Parks, Recreation and Conservation (retired), Ardsley, NY, pers. comm.). In marshes of the Hudson River and the New Jersey Meadowlands, larvae of *Poanes viator* (broad-winged skipper, a butterfly) feed on *Phragmites* leaves in the reedbeds, and the adults fly out of the reedbeds to feed on flower nectar of *L. salicaria* (purple loosestrife), *Nepeta cataria* (catnip), and other plants.

#### Comparisons of *Phragmites* and alternate habitats

I have compiled studies that compared the density (abundance) or taxon richness of invertebrates, fishes, and birds in *Phragmites* and alternate habitats (Tables 3–5). Density apparently varies by animal taxon, alternate habitat, environmental setting, season, survey method, and other factors. Table 3 shows comparisons of invertebrate assemblages in *Phragmites* and alternate habitats according to 20 studies. Most of these have been performed in tidal marshes of the East Coast, and most sampled nektonic or macrobenthic taxa. There is wide variation in the results of these heterogeneous studies, with density or richness less than, equal to or greater than that in *Phragmites;* however, *Phragmites* more often has lower density or richness than alternate habitats. Posey *et al.* (2003) found that the differences were due more to microtopography than to the plant *per se* for benthic invertebrates.

**Table 4:** Comparisons of fishes in *Phragmites* reedbeds vs. alternate habitat in US marshes. t, tidal marshes (various locations, East Coast); n, non-tidal marshes (Lake Erie, Ohio).

Fish (location)	Comparison	Alternate habitat	Source
*Fundulus heteroclitus* (mummichog) larvae and small juveniles (t)	Higher density in alternate habitat	*Spartina alterniflora* or *Typha*	Able and Hagan (2003); Able *et al.* (2003); Fell *et al.* (2003); Osgood *et al.* (2003, 2006); Raichel *et al.* (2003); Buchsbaum *et al.* (2006)
*F. heteroclitus* juveniles and adults (t)	Similar density in *Phragmites* and alternate habitat (also similar biomass in Rilling *et al.* 1998) (but lower density in *Phragmites* in Warren *et al.* 2001)	*Spartina alterniflora* or *Typha*	Rilling *et al.* (1998); Raposa and Roman (2001); Raichel *et al.* (2003); Fell *et al.* (2006)
*F. heteroclitus* eggs (t)	Similar abundance and development in *Phragmites* and alternate habitat	*Spartina alterniflora*	Able and Hagan (2003)
Species assemblage (t)	Similar density, species richness, or composition in *Phragmites* and alternate habitat	*Spartina alterniflora* or *Typha* (or various spp., Fell *et al.* 1998)	Fell *et al.* (1998, 2003); Meyer *et al.* (2001); Osgood *et al.* (2003); Raposa and Roman (2003); Buchsbaum *et al.* (2006); Fell *et al.* (2006)
Species assemblage (n)	Similar in *Phragmites* and alternate habitat	*Typha*	Kulesza (2006); Aday (2007)
Species assemblage (t)	Lower density in *Phragmites*	*Spartina alterniflora*, *Typha*	Warren *et al.* (2001)
Species assemblage (early life stages) (t)	Lower density in *Phragmites*	*Spartina alterniflora*	Able (1999)
*Fundulus luciae* (spotfin killifish) (t)	Present in alternate habitat but not in *Phragmites*	*Spartina alterniflora*	Able *et al.* (2003)
*Anguilla rostrata* (American eel), *Morone americana* (white perch) (t)	Higher density in *Phragmites*	Brackish meadow after *Phragmites* removal	Warren *et al.* (2001)

**Table 5:** Bird species that occur at higher or lower density in *Phragmites* vs. alternate habitats during breeding or non-breeding activities. All birds listed are native except European starling. Ca, *Carex atherodes*; Cc, *Calamagrostis canadensis;* Ce, *Carex emoryi;* Cg, *Carex gynandra*; Sch, *Scholochloa festucacea*; Sci, *Scirpus*; Sg, *Sparganium*; Sp, *Spartina*; SpA, *Spartina alterniflora*; SpP, *Spartina patens* and associated short graminoids; Ty, *Typha*. Location: Man., Manitoba; NS, Nova Scotia; Ont., Ontario; CA, California; CT, Connecticut; DE, Delaware; MA, Massachusetts; MD, Maryland; ME, Maine; NH, New Hampshire; NJ, New Jersey; NY, New York; RI, Rhode Island. A name and affiliation without a year indicate a personal communication.

Habitat; location	*Phragmites* greater	Alternate greater	Alternate plants	Source
*Breeding*				
Non-tidal wet meadow; Man.^a^	*Circus cyaneus* (northern harrier)		*Ty* etc.	Hecht (1951)
Non-tidal wet meadow; Man.	*Cistothorus platensis* (sedge wren), *Geothlypis trichas* (common yellowthroat)	*Xanthocephalus xanthocephalus* (yellow-headed blackbird), *Passerculus sandwichensis* (savannah sparrow), *Spizella pallida* (clay-colored sparrow)	*Sci, Sch, Ty*	Jones (1972)
Non-tidal marsh; Man.		*Sterna forsteri* (Forster's tern)	*Sci*	MacNicholl (1982)
Non-tidal wet meadow and marsh; Man.		Anatinae (dabbling ducks, 5 spp.)	*Sch*, other grasses	Sowls (1955)
Non-tidal experimental marshes; Man.		Anatidae (dabbling and diving ducks), *Fulica americana* (American coot)	*Ca, Sci, Sch, Ty*	Murkin *et al*. (1997)
Dredge spoil islands, estuaries; NJ	*Egretta thula* (snowy egret)*, Nycticorax nycticorax* (black-crowned night-heron), *Plegadis falcinellus* (glossy ibis)	Sternidae (terns), *Rhynchops niger* (black skimmer)	Bare sand	Kane (2001*a*); R. Kane, NJ Audubon Society
Dredge spoil island and marsh; DE^b^	*Plegadis falcinellus*	*Ardea herodias* (great blue heron), *A. alba* (great egret)	Shrubs and small trees	Parsons (2003)
Non-tidal marsh; NS^c^	*Podilymbus podiceps* (pied-billed grebe)	*Podilymbus podiceps*	*Sci, Sg, Ty*	Forbes *et al.* (1989)
Non-tidal marsh; Ont.	*Agelaius phoeniceus* (red-winged blackbird), *Dendroica petechia* (yellow warbler), *Geothlypis trichas*	*Aix sponsa* (wood duck)*, Botaurus lentiginosus* (American bittern), *Branta canadensis* (Canada goose)*, Cistothorus palustris* (marsh wren)*, Melospiza georgianus* (swamp sparrow)*, Podilymbus podiceps*, *Rallus limicola* (Virginia rail)*, Sturnus vulgaris* (European starling), *Turdus migratorius* (American robin)	*Cc, Ce, Cg*; *Ty*	Meyer (2003)
Non-tidal marsh; CA	*Rallus longirostris yumanensis* (Yuma clapper rail)^d^		*Sci,* some *Ty*	Anderson and Ohmart (1985)
Non-tidal riparian areas; Colorado River	*Geothlypis trichas*; total individuals, species richness		Many woody and herbaceous spp.	Spence (2006)
Fresh-tidal marsh; NY		*Cistothorus palustris*, *Agelaius phoeniceus*, *Melospiza georgiana*	*Ty*	Kiviat and Talmage (2006)
Fresh-tidal marshes; NY	Overall breeding season bird density and richness similar in *Phragmites* and alternate (small *Phragmites* stands)		Various	Mihocko (2001)
High salt marsh; NY, CT, MA, RI, NH, ME	*Botaurus lentiginosus*, *Cistothorus palustris*	*Ammodramus caudacutus s.l.* (sharp-tailed sparrows)*, Catoptrophorus semipalmatus* (willet)	*SpP, SpA*	Shriver and Vickery (2001); Shriver *et al.* (2004)
Tidal marshes; CT		*Rallus limicola*	*Ty*	Benoit and Askins (1999)
High salt marsh; CT		*Catoptrophorus semipalmatus*, *Ammodramus caudacutus* (saltmarsh sparrow), *A. maritimus* (seaside sparrow)	*SpP*	Benoit and Askins (1999)
Tidal marshes; MA	*Agelaius phoeniceus*	*Rallus limicola*, *Ammodramus caudacutus*	*Sp, Ty*	Holt and Buchsbaum (2000)
Tidal salt marsh; NJ^e^		*Larus atricilla* (laughing gull)	*SpA, SpP*	Burger and Shisler (1980)
*Non-breeding*				
Non-tidal riparian areas; Colorado River	*Geothlypis trichas, Thryomanes bewickii* (Bewick's wren); total individuals, species richness		Many woody and herbaceous spp.^f^	Spence (2006)
Fresh-tidal marsh; NY	Hirundinidae (swallows), Icteridae (blackbirds), *Sturnus vulgaris*		*Ty*	Kiviat and Talmage (2006)
Tidal marshes; MA	*Botaurus lentiginosus*, *Tachycineta bicolor* (tree swallow)		*Sp, Ty*	Holt and Buchsbaum (2000)
Tidal marshes; CT		*Rallus limicola*	*Ty*	Benoit and Askins (1999)
Brackish tidal marshes; RI		*Ardea alba, Egretta thula*^g^	*SpA*	Trocki and Paton (2006)

^a^All nests found in *Phragmites–Scholochloa festucacea* edges.

^b^These three species used a single habitat exclusively; four additional species nested in both *Phragmites* and woody vegetation. Among the latter group, cattle egret had greater nest success in *Phragmites*, little blue heron had greater success in upland woody vegetation, and snowy egret productivity varied.

^c^Nesting in patches dominated by the different plants was proportional to availability.

^d^One *Phragmites*-dominated marsh had the greatest density of rails compared with the expected value; two *Typha* marshes had high densities and one had a low density.

^e^Normally nested in *SpA* or less often *SpP*; in year of flood tides during April nested in *Phragmites*.

^f^Total individuals and species richness in breeding season related to total volume of annual plants, *Phragmites*, and *Phoradendron californicum* (mistletoe). In non-breeding season, *Thryomanes bewickii* related to *Phragmites* and *Acacia greggii* (catclaw); total individuals and species richness related to total annuals, *Phragmites*, and *Phoradendron californicum*.

^g^Egrets foraged in pools within *SpA* or *SpP* but not in *Phragmites* stands. *Phragmites* mostly occurred at the upland edges of the marshes.

Table 4 shows 16 fish studies that compared reedbeds with alternate habitats. Entire fish assemblages tend to be similar in *Phragmites* and *S. alterniflora* (or *Typha*) marshes or less dense in the *Phragmites*; in some cases the *Phragmites* marshes studied were tide restricted. However, the results for a small, abundant, and ecologically important tidal marsh fish *Fundulus heteroclitus* (mummichog) are different. Adult mummichogs are typically equally abundant in *Phragmites* and alternate plant communities, and spawn in both communities. Larval and small juvenile mummichogs are consistently less abundant in *Phragmites*. Raichel *et al.* (2003) hypothesized that young mummichogs were less abundant in *Phragmites* because of sparser prey resources. Although Osgood *et al.* (2006) found fewer juvenile mummichogs in *Phragmites* compared with *Typha*, this difference was apparently not related to benthic macroinvertebrate density or taxon richness. Weinstein *et al.* (2009) found lower levels of a biochemical indicator of condition, triacylglycerols, in *F. heteroclitus* from *Phragmites* compared with *S. alterniflora*. Dibble and Meyerson (2012) found that *F. heteroclitus* were healthier, as indicated by several morphological and physiological metrics, in tidally restored marshes with less *Phragmites* compared with tidally restricted marshes dominated by *Phragmites* in Rhode Island.

Raposa and Roman (2003) sampled three restricted–unrestricted marsh pairs where fish assemblages were less species rich with greater tide restriction; all restricted marshes were *Phragmites* dominated. Comparisons of fish assemblages in untreated *Phragmites* and herbicide-treated *Phragmites* have yielded variable results (Warren *et al.* 2001; Buchsbaum *et al.* 2006; Fell *et al.* 2006). In some cases the designs of nekton studies were confounded by elevation differences between *Phragmites* reedbeds and alternate habitats (e.g. Osgood *et al.* 2003), lack of measurement of elevation, or possibly hydrology and salinity rather than *Phragmites per se*.

Meyer (2003) found amphibian species richness to be similar in *Phragmites*, *Typha*, and ‘marsh meadow’ in non-tidal wetlands of Long Point (Lake Erie), Ontario, but a lower abundance in *Phragmites* compared with the alternate habitats. Also at Long Point, Bolton and Brooks (2010) documented rapidly spreading upland *Phragmites* overgrowing and detrimentally shading nest sites of freshwater turtles during incubation.

Relatively much is known about bird use of *Phragmites*, although this information is distributed unevenly by taxon, season, geographic region, and habitat (Table 5). In some cases, birds appear to actively select *Phragmites* habitat. Examples include *Sterna hirundo* (common tern) nesting in offshore reedbeds in Lake Poygan, Wisconsin (L. Bodensteiner, Western Washington University, USA, unpubl. data), *Oxyura jamaicensis* (ruddy duck) and *Fulica americana* (American coot) nesting only in reedbeds in New Jersey (Kane 2001*a*, *b*), and flocks of Hirundinidae (swallows), Icteridae (blackbirds), and other songbirds roosting in reedbeds in a freshwater tidal marsh on the Hudson River (Kiviat and Talmage 2006). In Maryland, blackbirds flew from as far away as 25 km to roost in reedbeds (Meanley 1993). Certain other species of birds have been found to avoid reedbeds, such as *Leucophaeus pipixcan* (Franklin's gull) at Bear River Migratory Bird Refuge in Utah (Olson 2007). Three species of conservation concern in Connecticut, *Catoptrophorus semipalmatus* (willet), *Ammodramus caudacutus* (saltmarsh sparrow), and *A. maritimus* (seaside sparrow), nested in the short graminoid meadows (*Spartina patens*, etc.) of the high salt marsh (Benoit and Askins 1999) and not in reedbeds. However, DiQuinzio *et al.* (2002), in nearby Rhode Island, found saltmarsh sparrow nesting in short *Phragmites* as well as in short native graminoids in a tidally restricted marsh. Although Sowls (1955) reported that nests of five species of Anatinae (dabbling ducks) were more common in alternate grass communities than in *Phragmites*, in the same wetland complex Ward (1942) stated that 31 % of 147 nests of ‘land-nesting’ ducks were in *Phragmites* edges. Ward considered water edges and wet meadow edges of reedbeds, mats of lodged culms in the water edges of reedbeds, small beds surrounded by wet meadow, and newly established, sparse reedbeds to be particularly favourable locations for duck nests. The difference between these two studies may have been due to the definition of reedbed edges or to reedbed management.

Of 17 studies of breeding birds in reedbeds compared with an alternate habitat (Table 5), there were about 16 instances of species that were more abundant in *Phragmites*, and about 36 instances of species more abundant in the alternate habitat (these tallies include some duplication of species among studies). Of six studies of non-breeding birds, there were about 13 instances of species that were more abundant in reedbeds and three instances of species more abundant in the alternate habitat. These numbers suggest that reedbeds offer more functions to non-breeding birds (e.g. cover for roosting and escape from predators), but the fact that >75 species of North American birds have been reported to be breeding in *Phragmites*-dominated habitat (some examples in Table 2) indicates the need for a broader range of studies. Meyer's (2003) study of birds in *Phragmites*, *Typha*, and marsh meadow at a Lake Erie site in Ontario indicated the complexity of *Phragmites*–bird relationships, which varied by habitat, stand edge compared with interior, season, and bird species. At a large and longstanding rookery on Pea Patch Island in Delaware Bay (Parsons 2003), two species of long-legged wading birds nested only in upland shrubs and trees, four species nested in that woody vegetation as well as in *Phragmites* marsh, and one species nested only in reedbeds. Of the four species that nested in both habitats, one had greater egg and nestling productivity in the reedbeds and one had greater productivity in the woody vegetation. Although alternate habitats may be better for more species, there are many cases where reedbeds are better for a particular species.

No bird that breeds in the U.S. or Canada is known to depend wholly on *Phragmites*, although certain birds breed only in *Phragmites* marshes in particular regions (e.g. *Fulica americana* and *Oxyura jamaicensis* in New Jersey (Kane 2001*a*, *b*). *Geothlypis beldingi* (Belding's yellowthroat, a wood warbler endemic to the oases of Baja California Sur, Mexico) breeds only in association with *Phragmites* reedbeds (Rodríguez-Estrella *et al.* 1999).

Although various species of small and large mammals have been reported using reedbeds (Table 2), few quantitative data are available. Meyer (2003) found greater abundance and richness of small mammals in *Phragmites* compared with *Typha* or marsh meadow in non-tidal wetlands of Long Point, Ontario. However, Meyer (2003) found white-tailed deer tracks to be more common in grass and sedge-dominated marsh meadow and *Typha* compared with *Phragmites. Peromyscus leucopus* (white-footed mouse) and the non-native *Mus musculus* (house mouse) frequented reedbeds in a Connecticut estuary, whereas *Microtus pennsylvanicus* (meadow vole) was more common in *Spartina patens* marsh (Holland and Smith 1980). McGlynn (2006) found the small-mammal species richness to be similar in *Phragmites* and two alternate habitats but *P. leucopus* more abundant in *Phragmites* in Hudson River fresh-tidal marshes.

*Phragmites* is used by many different organisms. In most cases it is not known whether these interactions are beneficial or detrimental to the species associated with *Phragmites.* In at least a few cases, *Phragmites* appears beneficial: roosting birds in reedbeds, songbirds eating seeds during migration or winter, animals taking refuge from flooding in reedbeds elevated above the surroundings, and small mammals like *Sylvilagus* (cottontails) hiding in reedbeds. In other cases, *Phragmites* appears detrimental: rapidly colonizing and shading turtle nesting sites, displacing the short graminoid community of high salt marsh on the northeastern coast, and supporting fewer young *F. heteroclitus* than in *S. alterniflora*-dominated wetlands. If it were possible to replace the *Phragmites* with fully functioning alternate habitats, would there be a real benefit to these species? Does the presence of reedbeds decrease the overall population of *F. heteroclitus*? The absence or scarcity of a species in a habitat does not necessarily mean that the habitat quality is poor (van Horne 1983). We need to understand the effects of *Phragmites* on a species at the levels of population and fitness, as well as the mechanisms of those effects, for each species. McGlynn (2006) found the body condition of small mammals, and mammalian predation on artificial songbird nests, to be similar in *Phragmites* and two alternate habitats. Parsons (2003) found the hatching success of *Egretta thula* (snowy egret) and *Egretta caerulea* (little blue heron) to be greater in woody vegetation and nestling survival of *E. caerulea* greater in woody vegetation, whereas the hatching success of *Bubulcus ibis* (cattle egret) was greater in reedbeds, and nestling survival of *E. thula* and *B. ibis* did not differ between habitats.

#### Reedbed characteristics and habitat functions

What makes a reedbed attractive to other organisms? The tall, dense masses of leafy culms where *Phragmites* is more highly dominant provide shelter from weather and predators to arthropods, small birds, and other small organisms, but may be too dense or shady for small plants or larger animals. However, large birds such as *Circus cyaneus* (northern harrier), Ardeidae (herons) and Threskiornithidae (ibises) can roost or nest on top of reedbeds with some degree of culm lodging. Large animals, such as *O. virginianus* (white-tailed deer), are sometimes able to break trails through dense reedbeds. Other *Phragmites* characteristics that shape its habitat functions include mats of lodged culms that animals rest on or under, hollow internodes of broken dead culms that shelter spiders, and the soil-stabilizing ability that apparently attracts *Castor canadensis* and *O. zibethicus* to build lodges. Some organisms are associated with high-biomass reedbeds whereas others are associated with low-biomass (sparse, short or fragmented) reedbeds.

**Figure 9.**
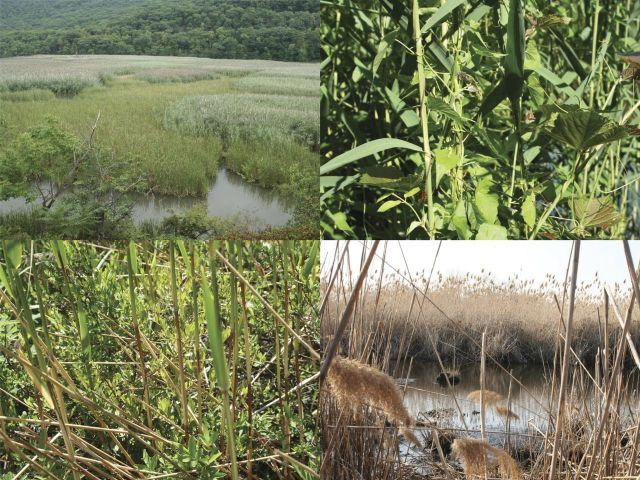
Variation in the reedbed habitat. Upper left: interspersion of *Typha* and Old World *Phragmites* in a brackish tidal marsh, Hudson River. Upper right: Old World *Phragmites* stand with the vines *Mikania scandens* (climbing hempweed) and *Ampelopsis brevipedunculata* (porcelainberry), New Jersey Meadowlands. Lower right: dense Old World *Phragmites* with small pool, New Jersey Meadowlands. Lower left: native *Phragmites* stand with an admixture of other plants, a marsh on Lake Ontario, New York. Photographs by Erik Kiviat.

The more we learn about how reedbed characteristics are beneficial or detrimental to particular species, the better we can manage *Phragmites* for particular biodiversity goals. It appears that extensive, dense beds of tall reeds support fewer species of breeding birds in the northeastern states than do small reedbeds, reedbeds with an admixture of other herbaceous or woody plants, sparse reedbeds and reedbeds in which patches of *Phragmites* are interspersed with pools or clearings (Fig. 9). Breeding season activity of *Gallinula chloropus* (common moorhen) in mine-associated wetlands was concentrated along reedbed edges and where reedbeds were interspersed with open water and abundant *Lemna* (duckweed) (Horstman *et al.* 1998). Nonetheless, *C. cyaneus* on the New Jersey and New York coast nested preferentially in dense, extensive reedbeds, although the same species in Manitoba nested in reedbed edges (Table 2). Meyer (2003) found greater abundance of birds in reedbed edges compared with interiors. Ward (1942) stated that few ducks nested in extensive dense reedbeds, but that small reedbeds and reedbed edges were highly selected. Possibly the edges of reedbeds are more attractive to foraging or nesting birds, as is often the case in *Typha* or other non-*Phragmites* wetland vegetation (Kostecke *et al.* 2004).

Litter, including lodged culms and culm stubble, affects animal use of reedbeds. *Turdus migratorius* (American robin) nested on mats of lodged culms (Hudson 1994). I observed *Sylvilagus* cf. *audubonii* in the Southwest using the same feature of reedbeds (Fig. 5).

Various intrinsic (stand) and extrinsic (environmental) variables may affect the suitability of *Phragmites* as habitat (Kiviat 2009*b*, 2010). Important intrinsic variables are reedbed extent and shape, the ratio of edge to interior, culm height and density, aboveground biomass, the ratio of fertile (flowering or fruiting) to sterile culms, lodging, litter mass and admixture (understory herbs, woody plants, vines, mosses). Important extrinsic variables are the presence of clearings or pools, soil microtopography and elevation, herbivory (beaver, muskrat, livestock, insects), surrounding land use and vegetation, human activities, proximity of other reedbeds, hydropattern (water levels, vertical and horizontal movement, and timing), soil texture and organic matter content, salinity, water quality, and the effects of ice, floods and fire. *Phragmites* reedbeds in tidal marshes tend to have more live biomass and litter mass, less microtopographic relief, and higher substrate elevation than the alternate plant communities such as *Spartina* spp. that *Phragmites* appears to replace (e.g. Meyerson *et al.* 2000; Angradi *et al.* 2001).

So far, there have been few studies comparing biodiversity support of different *Phragmites* haplotypes. Native haplotypes of *P. australis* tend to grow more sparsely with an admixture of other plants, compared with Old World *Phragmites* (E. Kiviat, unpubl. data). Differences in insect use of the subspecies were addressed by Lambert (2005).

The data summarized from many studies (Tables 2–5) indicate several possible generalizations about the biodiversity support services provided by *Phragmites*. There are many native and non-native species that occur in association with reedbeds. Some of these species are common in reedbeds, and some prefer reedbed edges while others also occur in reedbed interiors. In certain cases, mobile animals are clearly selecting reedbeds in landscapes containing alternate communities; roosting songbirds may be the best example. Because *Phragmites* (at least Old World *Phragmites*) tends to form dense stands with large amounts of live and dead biomass, many other vascular plants may not do well beneath the *Phragmites* canopies. In some cases, larger animals may have difficulty moving through reedbeds. None of these characteristics is unique to *Phragmites*; dense, low-diversity, high-biomass stands of *Typha*, *Scirpus*, tall *Carex* (sedge) species and other robust colonial marsh plants are similar in many respects.

#### Effects of other organisms on *Phragmites*

*Phragmites* is affected by many other non-human organisms; animals eat it, gall it, collect it for nest material and trample on it; taller plants shade it; fungi infect it; beavers flood it; and vines weigh it down. These interactions rarely seem to have a large impact. Probably the most common large effect of other organisms is due to *Ondatra* (muskrat) activities in cutting and excavating rhizomes and culm bases for food and lodge construction. Beaver activities may create habitat for *Phragmites* on abandoned dams and the bottoms of drawn-down abandoned ponds, but may also inhibit or kill *Phragmites* by flooding it. Although in many respects the reedbeds of today are relatively unaffected by the activities of other organisms, three types of interaction are worth noting: (i) sublethal effects that may alter productivity, reedbed architecture or other aspects of *Phragmites*; (ii) effects of prehistoric megafauna that may have been greater than animal effects seen now; and (iii) changing biotic interactions including the establishment of non-native species or increases in species already here.

Although many insects, non-native and native, feed on *Phragmites*, it is not generally regulated by insect herbivory (Balme 2000). However, I have seen local instances of significant damage to *Phragmites* patches by insect and muskrat herbivory. The muskrat, because it can reach high population densities, has the potential to inhibit or remove *Phragmites* in small and sometimes large areas; this activity can diversify reedbed vegetation. The combination of feeding on rhizomes and culm bases by muskrats and *Cyprinus carpio* (common carp) with high water levels and wind waves apparently caused recession of reedbeds in Kearny Marsh West in the Meadowlands in the early 2000s (E. Kiviat, unpubl. data). Muskrats, possibly in combination with insects or unidentified factors, fragmented a reedbed in South Glebe Marsh at Jug Bay Wetlands Sanctuary on the freshwater tidal Patuxent River in Maryland, and a few years later a vigorous but floristically diverse floating reedbed had developed at this site (E. Kiviat, unpubl. data). In the fen meadows of upper Moore Brook, Salisbury, Connecticut, *Phragmites* colonizing *Carex* meadows was alternately inhibited or facilitated by increases or decreases, respectively, in water levels caused by changes in beaver activity (E. Kiviat, unpubl. data).

*Nothrotheriops shastensis* (Shasta ground sloth, an extinct large mammal) fed intensively on *Phragmites* at an Arizona locality 40 000 years ago (Hansen 1978). *Mammuthus primigenius* (woolly mammoth, another Pleistocene megaherbivore) ate *P. australis* in Russia (Farrand 1961) and presumably did so in North America. Other Pleistocene large mammals, such as the *Castoroides* spp. (giant beavers) and many species of Equidae (horses), as well as Holocene mammals once much more abundant than now, such as *Bison bison* (American bison), may also have eaten *Phragmites*, as modern relatives do (Peden *et al.* 1974). Large animals such as these could have regulated or controlled *Phragmites* to an extent that is not seen now with wild mammals but is evident with livestock.

#### Is Old World *Phragmites* acquiring a biota in North America?

Non-native plants acquire a fauna of herbivorous insects in ∼30–200 years as a result of genetic adaptation of herbivores (Imura 1999; Carroll *et al.* 2005; Carroll 2007; Hawkes 2007) and possibly other processes. Non-native plants can also evolve to become less toxic to natural enemies and competitors (Lankau *et al.* 2009). The time required for adaptations to a non-native plant by users other than herbivorous insects has not been estimated. Old World *Phragmites* appears to be acquiring a biota after more than a century here. Many of the organisms that use *Phragmites* are generalists, excepting some of its insect herbivores. However, because native *Phragmites* is so similar to Old World *Phragmites*, organisms pre-adapted to using one should be able to switch relatively easily to the other.

There are several possible explanations for use of Old World *Phragmites* by any particular native species: (i) the species uses *Phragmites* as a result of exploratory behaviour or accident; (ii) *Phragmites* provides a low-quality resource where better habitat is not available, or is occupied by spillover (population pressure) from a better habitat that is saturated; (iii) a species is adapted to use non-native *Phragmites* because of pre-adaptation to native *Phragmites* or other tall graminoids; (iv) a species is an ecological generalist whose ‘partner’ range includes *Phragmites*; or (v) there has been recent evolutionary adaptation to increasingly widespread, abundant, robust, productive Old World *Phragmites* (it is also possible that Old World *Phragmites* has undergone recent evolution making it more suitable, e.g. more palatable, to a particular organism). Possibilities (iii) and (v) would result from fitness advantages gained by e.g. refuge from predation, harsh weather, human disturbance or another stressor. A species that encounters *Phragmites* by chance (option i) may eventually develop a more intimate relationship with *Phragmites*.

## Implications for management

Because *Phragmites* can provide substantial ecosystem services, as well as being a pest, it requires a management approach that is tailored to individual sites and sets of local management goals (Kiviat 2010). An approach that requires or encourages attempts to kill non-native taxa everywhere is impractical, causes non-target damage to sensitive species and wastes resources. The sanctity of native over non-native taxa has been challenged by, for example, Botkin (2001) and Cole *et al.* (2010). In order to focus efforts on situations where non-native taxa actually threaten sensitive native species or communities, it will be necessary to leave alone portions of stands or even entire stands of dominant non-native plants in certain situations. Although one plant community may support higher density or species richness than another, in most cases it is not richness *per se* that matters to nature conservation on a large scale, and it is more important to foster one or more species because it is a rare species or a resource (e.g. human food) species. In the case of *Phragmites*, I propose that the general management goal be the support of biodiversity through conservation of important species, balanced with promoting the ecosystem services and human uses provided by reedbeds. Specific goals should be set only after thorough biological surveys and realistic assessment of the long-term sustainability of any management action. Below I discuss problems with proposed and currently used management techniques and *Phragmites* research, and suggest future research directions.

### Problems with proposed biological control

Classical biological control, in which specialized natural enemies from the non-native plant's native range are introduced to the non-native range, is being developed for Old World *Phragmites* (Tewksbury *et al.* 2002; Blossey 2003). Once classical biocontrol organisms are released and established, they are intended to support themselves. Given the ecosystem services provided by *Phragmites*, there are several problems inherent in this approach. Biocontrol is likely to cause significant damage to reedbeds established for sewage sludge dewatering or nutrient removal from wastewater. Biocontrol is likely to cause the decline of, or alter the architecture of, reedbeds serving as habitat for marsh and water birds of conservation concern, and providing non-habitat ecosystem services such as stabilization and accretion of tidal marsh soils and carbon sequestration. Specialized natural enemies commonly switch hosts or may have host ranges broader than known; this phenomenon is well documented in insect herbivores (Strong *et al.* 1984; McEvoy and Coombs 2000). Biocontrol for *Phragmites* is intended to affect Old World *Phragmites* but not native *Phragmites* (Lambert 2005). Nonetheless, natural enemies specialized on Old World *Phragmites* are likely to switch to native *Phragmites*. The potential for host switching will put at risk all organisms that depend locally or regionally on native *Phragmites* for food or habitat, including *G. beldingi*, *Ochlodes yuma* (Yuma skipper) and any other *Phragmites*-dependent insects of western North America. The potential for loss of biodiversity is illustrated by the recent description of a new species of fly from native *Phragmites* in New York (Eichiner *et al.*, 2011). Furthermore, biocontrol has the potential to affect the ability of western and Mexican Native Americans to continue, or resume, using *Phragmites* in ceremonies, for ecological restoration, or for numerous other historical uses (see Kiviat and Hamilton 2001).

Once biocontrol is released and established widely, it cannot be ‘taken back’; the only way to protect reedbeds that provide valuable services would be to apply pesticides to kill the biocontrol organisms, and those pesticides could cause disruption of biodiversity and other services. Classical biocontrol, thus, would foreclose the option of managing reedbeds on a goal-directed and site-specific basis (Kiviat 2010; see below).

### Problems with other management techniques

More than any other technique, herbicides have been used to manage *Phragmites* (thousands of hectares in Delaware Bay alone), and most often the chemicals used have been glyphosate or one of its formulations. These herbicides have engendered genetic resistance in a number of weed species, and there is toxicological research indicating endocrine disruption, mutagenicity and carcinogenicity in animals (Kiviat 2009*a*). Even glyphosate used as a cut-culm treatment on *Phragmites* can leak into the environment and harm non-target plants (J. M. Toro, pers. comm.). Prescribed livestock grazing, although often effective for managing habitat for the endangered bog turtle (Tesauro 2001*a*, *b*), may harm certain non-target plant species. Mechanical control may also harm non-target plants. Removal of *Phragmites* by any technique may destabilize sediments, mobilize contaminants and result in marsh loss. All these techniques, nonetheless, have a place in the large-scale strategy of management.

### Research needs and problems

Although there has been much research on North American *Phragmites* in recent years, these studies have been affected by the methodological problems described here.

#### *Phragmites* morphology and reedbed architecture

The often dense tall culms impede observer vision and movement, and the abundant, silica-rich, standing dead culms make a loud noise when walked through. It is difficult to detect animals visually, estimate distances to those animals observed and move through reedbeds without scaring (or attracting) birds and other wildlife. Observer trails, and call playback used for surveying birds, can alter the behaviour of animals and cause them to move out of, or into, the reedbeds. These problems could be addressed through the use of small-scale remote sensing, including remote audio or video recording, camera traps, and possibly miniature remotely controlled aircraft (multicopters; Koch *et al.* 2011).

#### Genetic diversity

Native and non-native *Phragmites* are difficult to identify in the field, and in many cases genetic laboratory identification is necessary. Most studies cited here were conducted before genetic elucidation of *Phragmites* haplotypes, and voucher specimens of *Phragmites* apparently were not collected in most cases. As a result, we know little about the differences in ecological relationships between different *Phragmites* haplotypes.

#### Spatial and temporal bias

Most of the quantitative studies have been performed in tidal, rather than non-tidal, environments. For example, of 22 bird studies in Table 5, 15 were conducted in tidal environments. Almost all of the fish and invertebrate studies were conducted in tidal environments (Tables 3 and 4).

Reedbeds, and their biotic associates, are highly variable in space and time. It is necessary to sample widely to capture this diversity. Most of the quantitative studies reported here were performed in the New England or Middle Atlantic states. A few of the quantitative studies were performed before 1990. Most studies have used one or two study areas and sampled for 1 or 2 years. It is not known if the findings can be generalized to larger spatial and temporal scales.

#### Amount and location of information

Old World *Phragmites* is probably the most-studied non-native plant in North America. There is a large amount of information on *Phragmites* use by other organisms. Much of this information is qualitative, and much is in the grey literature or unwritten (see Table 2). Collecting and analysing this information is a formidable task, and I have probably compiled only a small portion of it in this paper.

#### General difficulties affecting studies of non-native organisms

Studies of *Phragmites* and other non-native plants in North America have typically begun with hypotheses of negative impacts on other organisms, potentially creating a bias in selecting research questions, study sites and methods, and interpreting results. In many cases, crucial habitat characteristics (e.g. substrate elevation in tidal marshes) have not been measured. Most studies have focused on relatively well-known groups of organisms (especially fishes, breeding birds, macrobenthic or nektonic estuarine invertebrates, and herbivorous insects) that may not represent ecological relationships of *Phragmites* with other taxa or guilds (taxonomic bias is also widespread with regard to rare species in conservation research and policy-making; Martín-López *et al.* 2009). Consideration of the biodiversity support functions of *Phragmites* has often been limited to food (e.g. for specialized herbivorous insects). Comparisons between biotas of *Phragmites* and alternate communities have almost always been based on population density (or catch-per-effort) or species richness, metrics that may not capture critical habitat functions of *Phragmites* for the most important species.

I have difficulty thinking of an a priori reason why a non-native plant should necessarily have a negative impact on a native species of animal or plant. There are many examples of non-native plants providing benefits to native organisms. One of the best documented is *Tamarix* (salt-cedar) as the breeding habitat for an endangered bird, *Empidonax traillii extimus* (southwestern willow flycatcher; Owen *et al.* 2005; Sogge *et al.* 2008). Another is the use of non-native larval host plants by a large number of butterfly species, benefiting certain species and harming others (Graves and Shapiro 2003). The concept of a priori neutrality is supported by many of the examples cited in this paper. Therefore I urge that researchers begin their studies of non-native plants with a null model (i.e. no differences compared with random).

#### Other experimental design considerations

Important environmental and stand variables should be measured or described as appropriate. In addition to breeding activities, the roosting and foraging activities of birds in various seasons need study. Other important groups needing study, in addition to fishes, estuarine invertebrates and herbivorous insects, include vascular plants, bryophytes, algae, mammals, reptiles, amphibians, terrestrial molluscs, butterflies, odonates (dragonflies and damselflies) and spiders. Species of conservation concern should receive priority attention, along with economically important species and keystone species or ecological engineers. Comparisons between *Phragmites* and alternate plant communities should consider, in addition to population density and species richness, functional metrics such as organism health (condition), diet, behaviour, reproduction and fitness. Guntenspergen and Nordby (2006) stated that experimental studies were needed to determine the impacts of *Phragmites* on terrestrial vertebrates of tidal marshes. Studies should include designs that examine responses of biota to experimental management of reedbeds, including partial removal of *Phragmites* biomass.

## Conclusions

*Phragmites*, and *Phragmites*-dominated habitats, support many ecosystem services and diverse native and non-native biota. Studies comparing the density of individuals or the numbers of taxa (species) in reedbeds and alternate habitats show variable results. Reedbeds apparently support fewer individuals or taxa of certain kinds of invertebrates, fishes and birds, such as early life stages of the mummichog, three species of high salt marsh breeding birds, and muskrat, than do alternate habitats.

Top-ranked food preferences, and relative density or taxon richness of breeding birds, monophagous herbivores and other groups, are not the only currency by which to judge *Phragmites*. Other important considerations include the rare species supported by reedbeds; the habitat functions of reedbeds for roosting, escape from predators and shelter from floods and other extreme conditions; the ability of *Phragmites* to vegetate urban habitats and derelict lands without human inputs; and other non-habitat ecological services provided by *Phragmites*. Given the severe changes in American landscapes and biotas resulting from land use, alteration of hydrology and chemistry, and climate change, concepts of the purity of native communities may not be practical for application to abundant, widespread, long-present non-native taxa such as Old World *Phragmites*. These arguments do not contravene controlling *Phragmites* where it is clearly a threat to important elements of biodiversity.

The use of *Phragmites* in wastewater management will continue to be important if it is not affected by biocontrol. *Phragmites* has good potential for bioenergy. The use of *Phragmites* fibre for paper, insulation and industrial materials should be explored. Given the new information presented here, we should look at *Phragmites* management as an optimization: how can we manage to increase and make use of the valuable ecosystem services provided by the plant, while reducing the harm that it causes in certain situations?

## Sources of Funding

My participation in the *Phragmites* Symposium of the 2011 Society of Wetland Scientists conference was partly funded by a grant from *AoB PLANTS* to the Smithsonian Institution, and by a donation to Hudsonia from the Kraus Dunne family. Previously, my research on *Phragmites* and its habitats has been funded by the Geraldine R. Dodge Foundation, Max and Victoria Dreyfus Foundation, Hudson River Foundation, Geoffrey C. Hughes Foundation, Metropolitan Conservation Alliance, New York City Environmental Fund, New York State Department of Environmental Conservation—Hudson River Estuary Program and Hudson River National Estuarine Research Reserve, and Westchester Community Foundation (all in the USA).

## Conflict of Interest Statement

Hudsonia is collaborating with TechnoPhrag, Inc. (Montréal, Québec) on a study of *Phragmites* fuel pellets; TechnoPhrag also offers *Phragmites* management services. Hudsonia also collaborates with Jason Tesauro Consulting LLC on biological survey and habitat management projects; in other projects this company manages *Phragmites* by means of prescribed livestock grazing. Hudsonia and the author do not have financial relationships either with entities that sell herbicides, mowing machinery or other supplies or equipment used for managing vegetation, nor with entities involved in research and development on biological control.
